# Glacier boundary extraction and spatiotemporal variation analysis in Geladandong region

**DOI:** 10.7717/peerj.20804

**Published:** 2026-02-18

**Authors:** Haotian Liu, Dongchuan Wang, Tingrong Li, Ang Yue, Shuang Zhao, Lihui Zhang, Kai Ye, Haotian Zhang, Shuaizheng Ji

**Affiliations:** 1 School of Geology and Geomatics, Tianjin Chengjian University, Tianjin, China; 2 Tianjin Key Laboratory of Aquatic Science and Technology, Tianjin, China; 3 Tianjin Eco-Environment Monitoring Center, Tianjin, China

**Keywords:** Glacier boundary extraction, Normalized difference indices and Random Forest, Normalized difference snow index, Normalized difference water index, Random Forests method, Geladandong region

## Abstract

Glaciers, as sensitive indicators of global climate change, play a crucial role in influencing the global water cycle, sea level rise, and ecosystem dynamics. Understanding the interactive mechanisms between glacier boundary changes and multidimensional factors such as climate and topography is essential for revealing the complex relationships underlying the ecological functions supported by glacier systems. This study proposes a Remote Sensing Index–Random Forest fusion method (NDI-RF) to map glacial extent. The NDI-RF approach combines remote sensing index techniques with Random Forest modelling, ensuring the extraction accuracy of the Random Forest model while effectively enhancing boundary extraction precision in mosaic pixel scenarios. Then, the spatiotemporal changes in glacier extent and their response to climate change were analyzed. The experimental results indicate that the NDI-RF method can reduce spectral confusion on ice lakes and thin ice surfaces to a certain extent. The extraction results show Kappa coefficient, OA, F1-score, recall, and Precision values of 0.92, 0.94, 0.92, 0.88, and 0.93, respectively, all of which outperform the Normalized Difference Snow Index (NDSI) extraction method and the Random Forest model. From 2000 to 2024, glaciers in the Geladandong region have mainly been shrinking in area, with a total reduction in glacier area of 110.29 km^2^ and an average annual area change rate of 0.47%. Among these years, the period from 2010 to 2015 was marked by the most significant glacier retreat, with a reduction of 36.87 km^2^, and also saw the highest glacier area change rate. Analysis based on different terrain conditions showed that glaciers retreated more notably at altitudes below 5,250 m, with slopes greater than 45°, and on north-west facing slopes. Over the past 25 years, the average annual temperature and total precipitation have shown a fluctuating upward trend. The glacier area shows a negative correlation with the average annual temperature.

## Introduction

Glaciers are highly sensitive to temperature variations and serve as a visible indicator of climate change, making them a vital component of the global climate monitoring network (Lorrey et al., 2022). Meanwhile, glaciers are vast and relatively stable solid water systems that store a large amount of fresh water and serve as natural freshwater reservoirs (Zhang et al., 2025a; Zhang et al., 2025b; Zeeshan et al., 2025). However, under the general environment of global warming, glaciers in various places generally show a trend of retreat (Korneva et al., 2024; Arora, 2020). The glaciers in the Geladandong region are located in the interior of the Tibetan Plateau. As critical headwater recharge zones for major rivers such as the Yangtze River and the Yellow River, these glaciers possess important ecological functions and scientific value. Variations in glacier extent directly influence the distribution of water resources and the stability of ecosystems across the Yangtze River Basin. Previous studies have documented glacier changes in the Geladandong region and have revealed a continuous reduction in glacier area from 1956 to 2012 (Chao et al., 2017). Glacier melt not only contributes to sea-level rise but also increases the risk of glacier lake outburst flood (GLOF), mudslides, and other natural hazards (He et al., 2024). In the Geladandong region, glacier retreat influences streamflow in rivers such as the Yangtze and Lancang, and can trigger glacial lake outburst floods and debris flows. These processes threaten the Tuotuo River Basin and adjacent transportation infrastructure. Consequently, research on glacier area change here is essential.

Glacier changes are governed by the combined effects of climate, topography, and other environmental factors (Zhao et al., 2024; Brun et al., 2019; Guha et al., 2025). Among these, topography has emerged as a key determinant in contemporary glacier research. Topographic parameters such as slope, aspect, and elevation strongly influence glacier accumulation, ablation, and flow dynamics (Guha, Tiwari & Zhang, 2025; Guha & Tiwari, 2022). These topographic factors introduce spatial heterogeneity in glacier response to climate change by modifying solar radiation receipt and snowline elevation—effects that have become increasingly pronounced under ongoing global warming (Fan, Tong & Ji, 2025; Zhang et al., 2024a; Zhang et al., 2024b; Li, Liu & Wu, 2021). Numerous studies have shown that glacier retreat is typically more intense at lower elevations, whereas high-altitude glaciers are often relatively stable (Han et al., 2025). Furthermore, slope and aspect influence surface energy balance and solar radiation distribution, thereby affecting rates of glacier shrinkage (Wang et al., 2024a; Wang et al., 2024b; Sun et al., 2025). In addition to topography, climatic drivers remain fundamental in shaping glacier change, as rising temperatures and variability in precipitation influence glacier mass balance and drive regional heterogeneity (Garg, Shukla & Jasrotia, 2019; Scherler, Bookhagen & Strecker, 2011). For instance, glaciers in the Karakoram are highly sensitive to climatic fluctuations: increased precipitation can promote glacier expansion, while rising temperatures cause substantial ablation, particularly in the eastern sector (Lhakpa, Fan & Cai, 2022). In the Kashmir Himalaya, glacier area has decreased by approximately 20% over the past three decades, closely linked to persistent climatic warming (Ahmad et al., 2021). Compared with the aforementioned regions, the Geladandong area, located in the interior of the Tibetan Plateau, experiences a climate that lies between the warm and humid southern slopes of the Himalayas and the cold, arid, and low-precipitation conditions of the Karakoram. This region is characterized by high elevation, low temperatures, and significant influence from summer monsoon precipitation. Therefore, the changes in its glaciers may fall somewhere in between, being constrained by topographic conditions and highly sensitive to climate fluctuations, and are expected to show a moderate-intensity retreat trend. This interplay of topography and climate provides a critical scientific basis for investigating the spatiotemporal patterns of glacier evolution in the Geladandong region.

Over the years, researchers have primarily employed visual interpretation, remote sensing index methods, and machine learning approaches for glacier boundary extraction. Visual interpretation relies on high-resolution imagery and expert knowledge (Man et al., 2014; Zhang et al., 2023). Although it achieves high accuracy, it is time-consuming, subjective, and difficult to apply to large-scale or long-term glacier monitoring. Remote sensing index methods (Zhang, 2024; Wilber et al., 2023), such as the Normalized Difference Snow Index (NDSI), enable automatic or semi-automatic extraction through sensitive band combinations. These methods offer good applicability, particularly in shadowed regions, but threshold selection is subjective, and they have limitations in handling mixed pixels. Machine learning approaches (Alifu et al., 2020; Ambinakudige & Intsiful, 2022; Shikha et al., 2022; Sharma et al., 2022), including Support Vector Machines and Random Forests, can improve extraction efficiency but are highly dependent on sample quality. Given the spectral similarity between debris-covered ice and surrounding rocky surfaces, traditional index-based methods often produce boundary confusion (Pandey et al., 2012; Guha & Tiwari, 2022). Additionally, surfaces of some glacial lakes covered with broken or thin ice may introduce mixed-pixel interference. To address these challenges, this study proposes the Remote Sensing Index Random Forest fusion (NDI-RF) method, building upon previous approaches. By incorporating the NDSI (Worni, Huggel & Stoffel, 2013; Chen et al., 2021; Wang et al., 2020a; Wang et al., 2020b) and Normalized Difference Water Index (NDWI) (Wendleder, Friedl & Mayer, 2018), the method establishes a comprehensive identification framework for snow, ice, and water, allowing for the removal of mixed pixels with strong water components and thereby improving boundary purity. Subsequently, multi-source features including spectral data, texture and topography were input into a Random Forest model for classification optimisation, thereby achieving more stable glacier boundary extraction within debris-covered areas and complex shadow zones.

Accordingly, this study utilizes remote sensing imagery from the Landsat satellite series and employs the NDI-RF method to accurately extract glacier boundaries. Based on the extracted glacier boundaries, the spatiotemporal variations of glaciers in the Geladandong region from 2000 to 2024 are analyzed across three topographic dimensions: elevation, slope, and aspect. Furthermore, using meteorological data from the Tuotuohe station for the period 1995–2019, the study explores in depth the response mechanisms of glaciers in the Geladandong region to climate change. The findings provide a scientific basis for the conservation of glacier resources in the source region of the Yangtze River and offer multi-dimensional decision-making support for regional ecological sustainability and the refinement of climate models.

## Materials and Methods

### Overview of the study area

**Figure 1:**
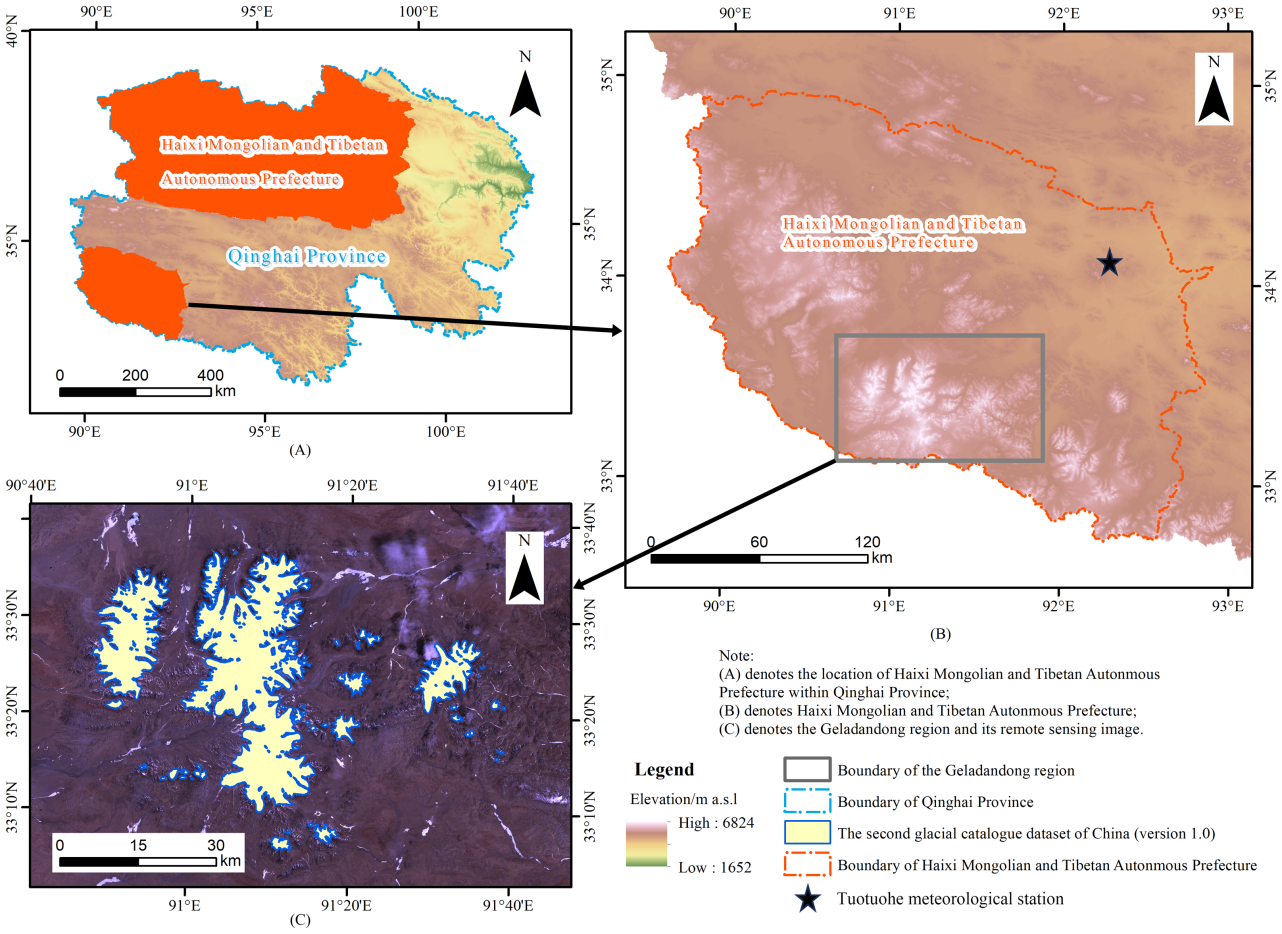
Location map of the study area. (A) The location of Haixi Mongolian and Tibetan Autonmous Prefecture within Qinghai Province; (B) Haixi Mongolian and Tibetan Autonmous Prefecture; (C) the Geladandong region and its remote sensing image.

The Geladandong region is located in the central part of the Qinghai-Tibet Plateau, along the northern section of the Tanggula Mountains (Fig. 1). It is one of the source areas of important rivers such as the Yangtze and Lancang Rivers and is a typical high-altitude glacier development area. The climate in this region is cold and dry, with year-round temperatures below 0 °C, and precipitation mainly occurs in the summer, with an annual precipitation of about 200 mm (Zhang et al., 2014). In recent years, under the background of global climate warming, the temperature in this region has shown a fluctuating upward trend, leading to the continuous retreat of glaciers. In some glacier ablation zones, distinct debris-covered features have emerged (Chao et al., 2017).

### Data source

The remote sensing imagery used in this study includes Landsat-5 TM and Landsat-8 OLI data, with a spatial resolution of 30 m. These images were obtained from the Geospatial Data Cloud platform (http://www.gscloud.cn/) and the Earth Explorer platform of the United States Geological Survey (USGS) (https://earthexplorer.usgs.gov/). A total of eight scenes were selected, corresponding to the years 2000, 2005, 2007, 2010 (two scenes), 2015, 2020, and 2024. Since autumn occurs at the end of the glacier ablation season, there is little seasonal snow accumulation on the glacier surface, and the glacier boundaries are clear. Therefore, the imagery collection period is mainly concentrated in autumn (September to November). For further details, please refer to Table 1. The Second Glacial Catalogue Dataset of China (Version 1.0) was obtained from the National Cryosphere Desert Data Center (https://www.ncdc.ac.cn). In this dataset, the most recent glacier vector data for the study area is from 2007. Hence, this study selects the 2007 data for the experiments and verifies the results against the existing data in the Second Glacial Catalogue of China. The Digital Elevation Model (DEM) data were derived from NASA DEM, provided by the National Aeronautics and Space Administration (NASA) (https://www.nasa.gov/). Meteorological data were sourced from the China Meteorological Data Service Centre (https://data.cma.cn/). Due to the remoteness of the Geladandong region and the absence of *in-situ* meteorological stations, the Tuotuohe station—the closest meteorological station to the study area—was selected to represent the regional climate background. The meteorological data span the years 1995 to 2019. The specific data sources are shown in Table 2.

### Methods

#### Normalized difference snow index

NDSI is widely recognized for its efficiency, accuracy, and broad applicability. However, it may occasionally misclassify surface features with spectral characteristics similar to snow and ice (Raghubanshi, Agrawal & Rathore, 2023). In NDSI imagery, snow and ice typically exhibit high positive values, while other land covers tend to display low or negative values. The NDSI is calculated using the following formula:

(1) \begin{eqnarray*}\mathrm{NDSI}= \frac{\mathrm{Green-SWIR}}{\mathrm{Green}+\mathrm{SWIR}} .\end{eqnarray*}

**Table 1:** Remote sensing imagery data.

Sensor type	Grade	Imaging date	Path/Row	Cloud (%)
Landsat-5 TM	L1	2000.10.08.	138/37	1.62
Landsat-5 TM	L1	2005.10.06.	138/37	8.71
Landsat-5 TM	L1	2007.09.26.	138/37	6.13
Landsat-5 TM	L1	2010.10.20.	138/37	9.87
Landsat-5 TM	L2	2010.11.05.	138/37	8.14
Landsat-8 OLI	L1	2015.10.02.	138/37	0.24
Landsat-8 OLI	L2	2020.10.31.	138/37	0.85
Landsat-8 OLI	L2	2024.10.10.	138/37	4.30

**Table 2:** Data sources.

Data type	Data name	Resolution	Data sources
Remote sensing image	Landsat-5 TM	30 m	Geospital Data Cloud platform (http://www.gscloud.cn/)
	Landsat-8 OLI	30 m	United States Geological Survey (USGS) (https://www.usgs.gov)
Glacial	The second glacial catalogue dataset of China (version 1.0)	30 m	National Cryosphere Desert Data Center (https://www.ncdc.ac.cn)
DEM	NASA DEM	30 m	National Aeronautics and Space Administration (https://www.nasa.gov/)
Meteorological data	Temperature Prepicitation	–	China Meteorological Data Service Centre (https://data.cma.cn/)

In the above formula, Green refers to the green band of the imagery, and SWIR refers to the shortwave infrared band. For Landsat-5 TM, these correspond to Band 2 and Band 5, while for Landsat-8 OLI, they correspond to Band 3 and Band 6, respectively. Based on previous studies on NDSI (Jara et al., 2024), and considering the actual conditions of the study area, the threshold for NDSI was set to 0.5.

#### Normalized Difference Water Index

The NDWI is a commonly employed index for water body extraction (Liu et al., 2025a; Liu et al., 2025b). Water bodies typically exhibit high positive values in NDWI imagery, whilst other land features—such as vegetation, soil, and structures—display lower values or negative values. The NDWI is calculated using the following formula:

(2) \begin{eqnarray*}\mathrm{NDWI}= \frac{\mathrm{Green-NIR}}{\mathrm{Green}+\mathrm{NIR}} .\end{eqnarray*}

In the above formula, Green refers to the green band of the imagery, and NIR refers to the near-infrared band. For Landsat-5 TM, these correspond to Band 2 and Band 4, while for Landsat-8 OLI, they correspond to Band 3 and Band 5, respectively. Based on previous studies on NDWI (Wendleder, Friedl & Mayer, 2018; Prateek & Raaj, 2022), and considering the actual conditions of the study area, the threshold for NDWI was set to 0.1.

#### Random Forest model

The Random Forest (RF) algorithm is a traditional algorithm in the field of machine learning. It can generate multiple sample subsets from the original training set through random sampling with substitution, and independently construct decision tree models. The decision trees do not affect each other and can be trained and predicted in parallel. It has a high tolerance for outliers that occur during the image extraction process, making it more convenient and efficient in practical applications. The Random Forest algorithm effectively avoids interference from noisy samples, enhancing the model’s stability and reliability. It achieves fast extraction speeds while maintaining good extraction accuracy (Azar, Raul & Emma, 2019). The specific formula is as follows:

(3) \begin{eqnarray*}\mathrm{H} \left( \mathrm{x} \right) ={\mathrm{argmax}}_{\mathrm{y}}\sum _{\mathrm{i}=1}^{\mathrm{k}}\mathrm{I} \left( {\mathrm{h}}_{\mathrm{ i}} \left( \mathrm{x} \right) =\mathrm{Y} \right) .\end{eqnarray*}

In the formula, H(x) denotes the final output result, hi represents the individual decision tree classifiers, Y is the output variable, and I(x) is the indicator function.

In this study, a stratified random sampling strategy was used to select training and testing data points. First, based on clearly defined glacier boundaries (from The Second Glacial Catalogue Dataset of China) and non-glacial areas, multiple representative training regions were manually delineated. Then, within these predefined training regions, independent training and testing data points were generated using random sampling, with a 7: 3 ratio for dividing the training and testing sets. On average, the number of sample points per year was 815. Referring to previous studies (Lu, Zhang & Huang, 2020; Liu et al., 2025a; Liu et al., 2025b), and after multiple preliminary experiments, the number of decision trees was finally determined to be 300.

#### Feature selection

Given the large topographic relief and the presence of debris-covered glaciers in the Geladandong region, the input features for the Random Forest model were comprehensively selected from three categories—spectral (Wu, Ren & Wang, 2022; Feng et al., 2023; Wang et al., 2024a; Wang et al., 2024b), textural (Wang et al., 2022; Yang et al., 2023), and topographic features (Lu, Zhang & Huang, 2020; Luo et al., 2024)—to enhance the model’s discriminative capability under complex surface conditions. The spectral features were derived from atmospherically corrected Landsat image bands; the textural features were extracted using the Gray-Level Co-occurrence Matrix (GLCM) within a local moving window to characterize surface structural variations; and the topographic features were calculated from DEM data, including elevation, slope, and aspect. To ensure consistency among the input features, all data were resampled to a 30 m spatial resolution, matching the main Landsat bands. Regarding spectral features, they generally include both band-based and index-based characteristics. Prior to the application of the Random Forest model, NDSI and NDWI were introduced for preliminary glacier extraction and mixed-pixel removal. To avoid information redundancy and multicollinearity with the previously used index variables, only the original spectral bands were selected as input features for the Random Forest model at this stage, aiming to further optimize glacier boundary extraction. For specific feature information, please refer to Table 3.

**Table 3:** Feature selection.

Input parameters	Feature selection
Spectral features	1.For Landsat-5, six spectral bands were selected: Blue, Green, Red, NIR, SWIR1, and SWIR2. 2. For Landsat-8, seven spectral bands were used: Blue, Green, Red, NIR, SWIR1, SWIR2, and TIRS2.
Textural features	1. Mean. 2. Contrast. 3. Correlation. 4. Variance. 5. Angular Second Moment,(ASM). 6. Entropy. 7. Homogeneity. 8. Dissimilarity.
Topographic features	1. Elevation. 2. Slope. 3. Aspect. 4. Shaded Relief. 5. Plan Convexity. 6. Profile Convexity.

#### Remote Sensing Index-Random Forest fusion method (NDI-RF)

**Figure 2:**
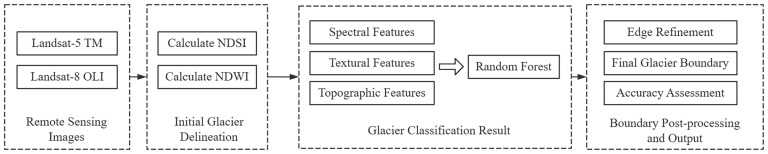
NDI-RF flowchart.

This study developed a glacier identification method that integrates remote sensing indices with machine learning (NDI-RF). In high-mountain glacier regions, some glacial lakes may be partially covered by broken ice or thin ice, causing their spectral signatures to similar toward those of snow or debris-covered ice (Hu et al., 2022), which can easily lead to confusion during Random Forest classification. To further eliminate the influence of such mixed pixels, this study introduces the NDWI index for auxiliary elimination. Rather than serving to identify liquid water bodies in the conventional sense, NDWI is employed as a supplementary indicator to detect and remove mixed pixels with significant water components, thereby improving the purity of glacier boundaries (Fig. 2).

First, an initial glacier mask was generated. The Normalized Difference Snow Index (NDSI), constructed using the ratio between the green and shortwave infrared bands, exhibits high spectral sensitivity to snow and ice. Accordingly, NDSI was applied to Landsat imagery to derive the initial glacier mask. The purpose of this step is to perform a binary classification of ice *versus* non-ice, thereby obtaining a preliminary distribution of glacier extent rather than directly delineating glacier boundaries. Second, NDWI was introduced to remove interference. Pixels with NDWI values exceeding a given threshold were excluded from the initial glacier mask, effectively suppressing the influence of mixed pixels on glacier boundary extraction and yielding an initial delineation of glacier extent. Nevertheless, the preliminary boundaries remain affected by factors such as cast shadows and fragmented ice zones. Therefore, further optimization was performed through multi-feature classification: spectral, textural, and topographic features were jointly input into a Random Forest model to refine classification results. The final glacier boundaries were then subjected to edge optimization to improve overall shape integrity.

#### Accuracy evaluation metrics

Accuracy evaluation metrics are numerical indicators used to assess the precision of extraction results and serve as an important basis for quantitative analysis. This study employed the Kappa coefficient, Overall Accuracy (OA), F1-score, Recall, and Precision for accuracy assessment.

(4) \begin{eqnarray*}\mathrm{k}& = \frac{{\mathrm{P}}_{0}-{\mathrm{P}}_{\mathrm{e}}}{1-{\mathrm{P}}_{\mathrm{e}}} \end{eqnarray*}

(5) \begin{eqnarray*}{\mathrm{P}}_{0}& = \frac{\mathrm{TP}+\mathrm{TN}}{\mathrm{TP}+\mathrm{FP}+\mathrm{FN}+\mathrm{TN}} \end{eqnarray*}

(6) \begin{eqnarray*}{\mathrm{P}}_{\mathrm{e}}& = \frac{ \left( \mathrm{TP}+\mathrm{FP} \right) \left( \mathrm{TP}+\mathrm{FN} \right) + \left( \mathrm{FN}+\mathrm{TN} \right) \left( \mathrm{FP}+\mathrm{TN} \right) }{{ \left( \mathrm{TP}+\mathrm{FP}+\mathrm{FN}+\mathrm{TN} \right) }^{2}} \end{eqnarray*}

(7) \begin{eqnarray*}\mathrm{OA}& = \frac{\mathrm{TP}+\mathrm{TN}}{\mathrm{TP}+\mathrm{TN}+\mathrm{FP}+\mathrm{FN}} \end{eqnarray*}

(8) \begin{eqnarray*}\mathrm{F}1& = \frac{2\times \mathrm{TP}}{2\times \mathrm{TP}+\mathrm{FP}+\mathrm{FN}} \end{eqnarray*}

(9) \begin{eqnarray*}\mathrm{Recall}& = \frac{\mathrm{TP}}{\mathrm{TP}+\mathrm{FN}} \end{eqnarray*}

(10) \begin{eqnarray*}\mathrm{Precision}& = \frac{\mathrm{TP}}{\mathrm{TP}+\mathrm{FP}} .\end{eqnarray*}

In the formulas, k represents the Kappa coefficient, OA denotes the Overall Accuracy, and F1 stands for the F1-score. P_*o*_ refers to the observed agreement (Overall Accuracy), which is the proportion of agreement between the classification results and the reference data. P_*e*_ represents the expected agreement (Expected Accuracy), which is the proportion of agreement that could occur by chance. Recall refers to the ratio of the number of pixels correctly classified as glacier to the total number of glacier pixels in the reference data. Precision refers to the ratio of the number of pixels correctly classified as glacier to the total number of pixels that the classifier labeled as glacier in the entire image. TP (True Positive) indicates the area correctly identified as glacier in both the extraction results and the reference data. FP (False Positive) refers to the area identified as glacier in the extraction results but classified as non-glacier in the reference data. FN (False Negative) denotes the area classified as non-glacier in the extraction results but identified as glacier in the reference data. TN (True Negative) represents the area correctly identified as non-glacier in both the extraction results and the reference data. Specifically, in this study, P_*e*_ is calculated based on the row and column distributions of the confusion matrix to estimate the probability that the classification results agree with the reference data under purely random conditions. The calculation is as follows:

(11) \begin{eqnarray*}{\mathrm{P}}_{\mathrm{e}}=\sum _{\mathrm{i}=1}^{\mathrm{n}} \left( \frac{{\mathrm{R}}_{\mathrm{i}}\times {\mathrm{C}}_{\mathrm{i}}}{{\mathrm{N}}^{2}} \right) .\end{eqnarray*}

Here, *R*_*i*_ is the number of reference samples in class *i*, *C*_*i*_ is the number of samples classified as class *i*, and *N* is the total number of samples.

## Results and Analysis

### Glacier boundary extraction

#### Extraction results and accuracy validation

**Figure 3:**
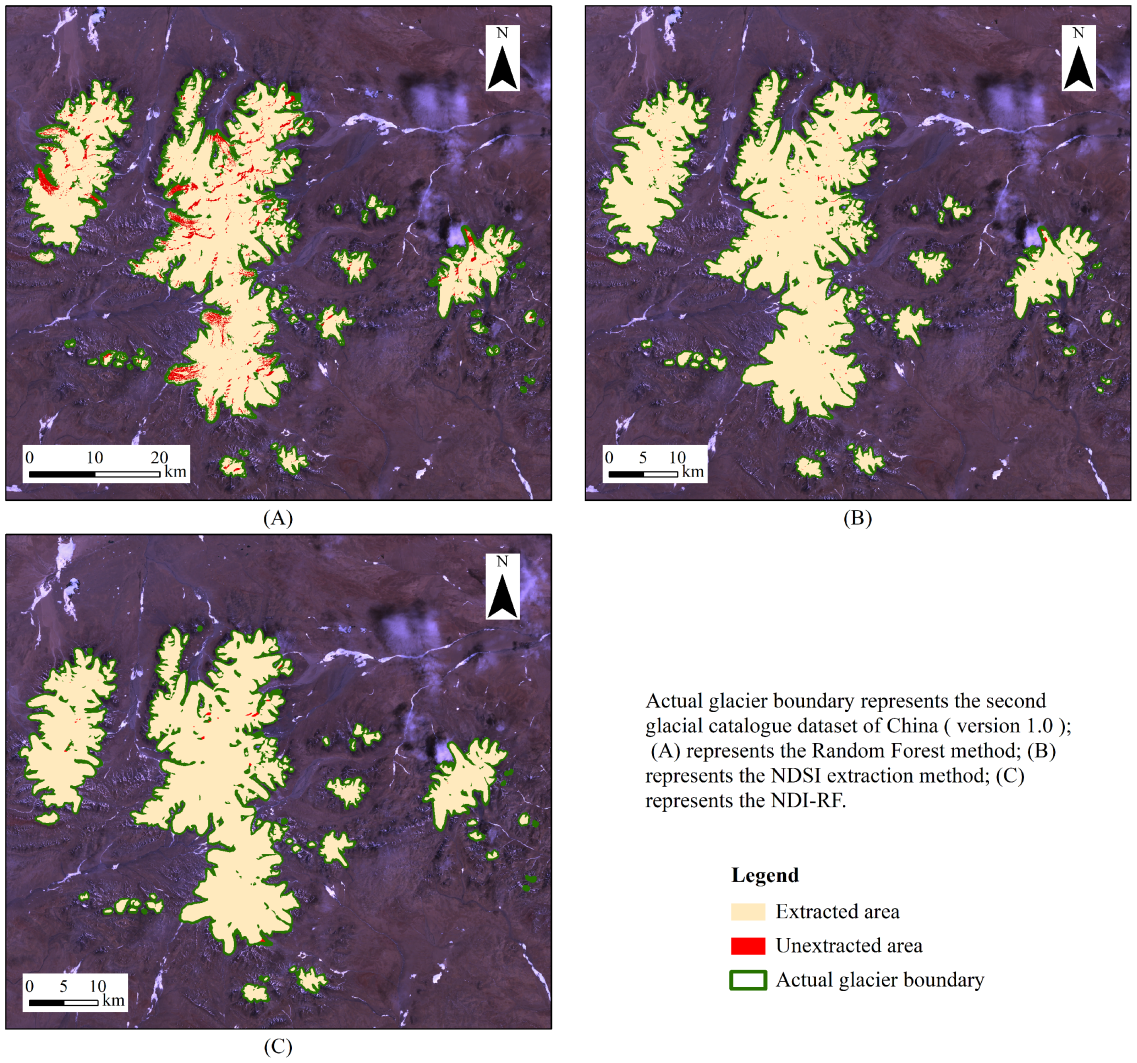
Glacier extract results by various methods (2007). Actual glacier boundary represents the second glacial catalogue dataset of China (version 1.0); (A) the Random Forest method; (B) the NDSI extraction method; (C) the NDI-RF.

Based on Landsat satellite imagery, this study extracted glacier boundaries using the NDI-RE method and obtained glacier delineation results for the study area in 2007. The results were compared with those derived from the NDSI method and the Random Forest algorithm. As shown in Fig. 3, the glacier boundaries extracted by the NDI-RE method demonstrated high accuracy. To further evaluate the consistency between the extraction results and the reference dataset, the Kappa coefficient, OA, F1-score, Recall, and Precision were used to assess the accuracy of three methods. As shown in Table 4, the NDI-RF method achieved a Kappa coefficient, OA, F1-score, Recall, and Precision of 0.92, 0.94, 0.92, 0.88, and 0.93, respectively, indicating high extraction accuracy. Compared with traditional index-based methods and single classification models, the NDI-RF method, which combines index-based pre-screening with Random Forest classification, not only maintains a leading overall accuracy but also demonstrates superior reliability in terms of boundary continuity and classification stability.

**Table 4:** Kappa, OA, F1-score, Recall and Precision results by methods.

Methods	Kappa coefficient	OA	F1-score	Recall	Precision
Random Forest	0.9072	0.9279	0.9143	0.87	0.91
NDSI	0.8892	0.9083	0.8991	0.86	0.90
NDI-RF	0.9179	0.9376	0.9246	0.88	0.93

#### Analysis of spatiotemporal variation characteristics of glaciers

As shown in Table 5, the glacier retreat rates in the Geladandong region between 2000 and 2024 exhibits significant stage-specific differences. Overall, the retreat was not uniform but showed strong fluctuations. The period from 2010 to 2015 represents the peak of glacier area change, with both the area change and the rate of area change far exceeding those of other periods, indicating that climatic or other environmental factors exerted an especially strong driving effect on glacier ablation during this time. In contrast, the period from 2000 to 2005 shows the slowest glacier area change. Notably, after the peak area change, the area change rate slowed during 2015–2020, but accelerated again from 2020 to 2024, even surpassing the level observed in 2005–2010. This indicate that glacier area change in the study area has entered a new phase of acceleration. This unstable pattern indicates that the dynamic changes of glaciers in the Geladandong region are a nonlinear response resulting from the combined effects of climate fluctuations and complex topography.

**Table 5:** Characteristics of glacier area changes in the study area by time period.

Year	Area/km^2^	Glacier area change/km^2^	Rate of change/%	Change rate/(km^2^/a)
2000	941.61	–	–	–
2005	931.61	−10.00	1.06	−2.00
2010	906.73	−24.88	2.67	−4.98
2015	869.86	−36.87	4.06	−7.37
2020	852.22	−17.64	2.02	−3.53
2024	831.32	−20.90	2.45	−4.18

**Figure 4:**
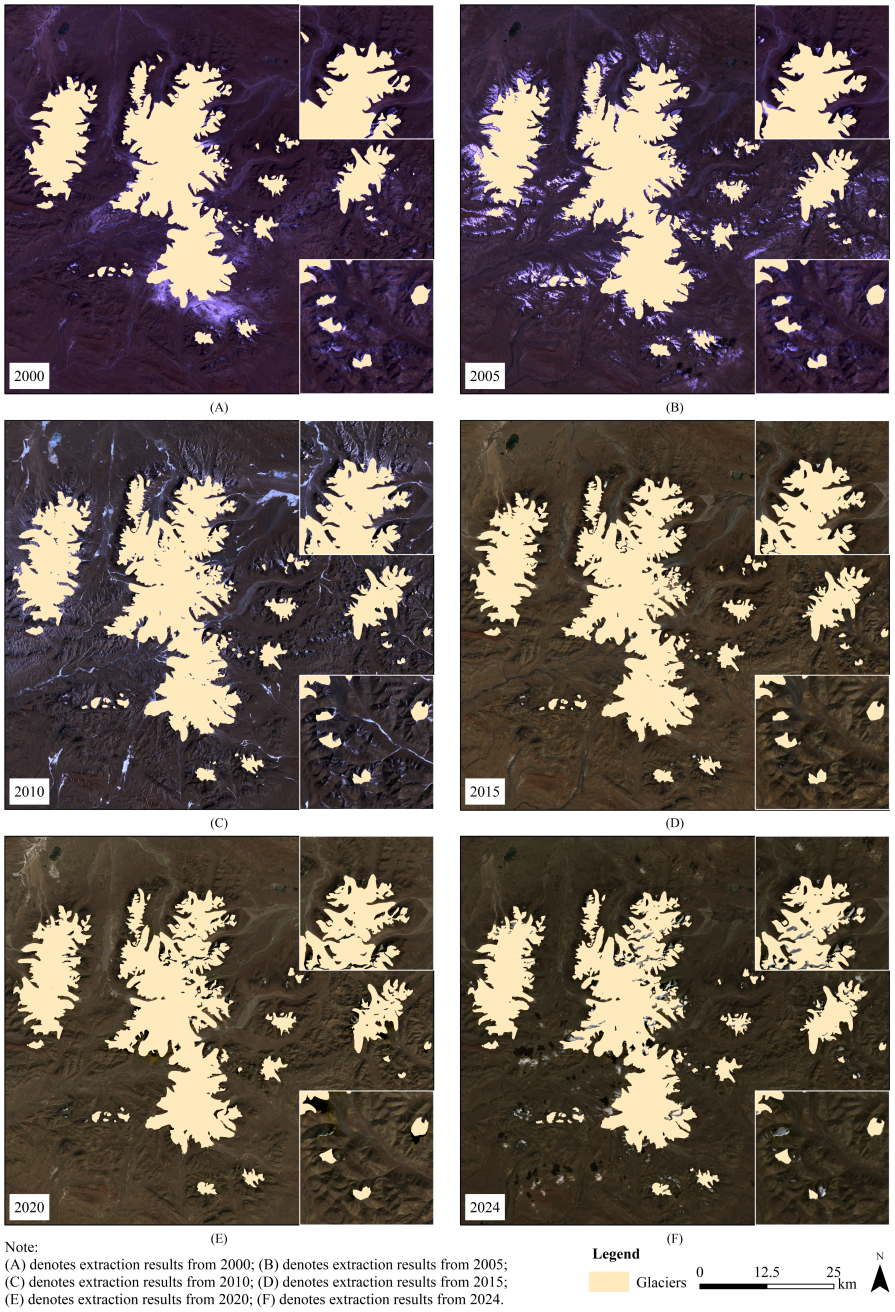
Distribution of glacier boundaries 2000-2024. (A–F) The extract results for the years 2000, 2005, 2010, 2015, 2020, and 2024, respectively.

As illustrated in Fig. 4, between 2000 and 2024, the overall glacier extent has decreased, with notable retreat observed in specific regions. In Region I, the glaciers primarily terminus retreat. The glaciers’ termini extend into relatively low-elevation valleys, making them particularly sensitive to rising temperatures. Intense ablation has caused the ice tongues to retreat continuously, rendering the glaciers margin fragmented and irregular, forming isolated ice bodies that accelerated disappearance. In Region II, the glaciers typically displays the dramatic response of small glaciers. The glaciers in this region were relatively small in scale and had limited ice reserves, which results in a much weaker buffering capacity against climate change compared to larger glaciers. Under the continued warming climate, these small glaciers not only experience rapid terminus retreat but also undergo drastic reductions in surface area, with some glaciers completely disappearing during the observation period. In 2000, the glaciers were generally more intact, with no significant fragmentation. The glacier fronts were situated at relatively low elevations, extending into valleys or plains. Over the past 25 years, however, fragmentation has gradually intensified, and the glacier fronts have steadily retreated to higher elevations. This trend is driven by rising temperatures, which accelerate melting at the glacier termini, while glacier flow is unable to replenish the lost ice in time, resulting in the progressive retreat of the glacier fronts (Nie et al., 2024). Overall, during the past 25 years, glacier area changes had varied across different regions of the study area. The glaciers as a whole have exhibited significant retreat, with some small glaciers even disappearing entirely.

Figures 4A–4F represent the extract results for the years 2000, 2005, 2010, 2015, 2020, and 2024, respectively.

### Analysis of glacier change characteristics under different topographic conditions

Changes in glacier area are influenced not only by climatic factors such as temperature and precipitation, but also by topographic elements, which play a crucial role in the spatial variation of glacier extent (Remya et al., 2022; Brun et al., 2019). Therefore, analyzing glacier area changes under different topographic conditions enables a more in-depth understanding of glacier spatial dynamics. Based on previous studies (Han et al., 2025; Wang et al., 2024a; Wang et al., 2024b; Sun et al., 2025) and the specific characteristics of the study area, elevation, slope, and aspect were categorized into several intervals for detailed analysis.

#### Analysis of spatial variation characteristics of glaciers at different elevations

As shown in Table 6, the dynamic changes in glacier area from 2000 to 2024 across different elevation ranges are presented. Overall, the rate of glacier area change exhibits a nonlinear distribution pattern of “high at both ends and low in the middle”. Specifically, the glacier area change rate first decreases and then increases with elevation, reaching its minimum at 5,500–6,000 m. Specifically, area below an elevation of 5,500 m experienced the largest amount of glacier area changes. Among them, glaciers below 5,250 m have completely disappeared, while those in the 5,250–5,500 m elevation range have decreased in area by 46.44%. This was because this elevation range lies in the lower part of the glacier ablation zone, where the impact of temperature is most pronounced. The large rate of area change also confirmed that this zone was highly sensitive to rising temperatures. The glacier area change rate is lowest in the 5,500–6,000 m elevation zone, which correlates with the relatively low temperatures and stable snow accumulation capacity at these altitudes. It is noteworthy that glaciers located above 6,000 m did not show a continuous decreasing trend in their area change rate; instead, a rebound is observed. This indicates that the response mechanisms of glaciers in this elevation range to climate change were more complex, suggesting that glacier stability did not increase linearly with elevation.

**Table 6:** Statistical table of glacier area (m^2^) at different elevations.

Year	Elevation/m						
	<5,250	5,250–5,500	5,500–5,750	5,750–6,000	6,000–6,250	6,250–6,500	>6,500
2000	37.66	7,730.01	41,806.77	37,164.64	6,585.25	818.20	18.83
2005	18.63	6,190.17	41,550.90	37,752.95	6,791.40	838.44	18.63
2010	27.20	6,123.27	40,109.43	36,930.02	6,641.3	824.12	18.13
2015	0.00	5,532.29	38,647.75	35,690.23	6,315.16	782.87	17.40
2020	0.00	4,976.99	38,222.25	35,299.12	6,025.22	681.78	17.04
2024	0.00	4,139.95	38,448.37	34,017.45	5,819.21	689.99	16.63
2000–2024 area change rate/%	100.00	46.44	8.03	8.47	11.63	15.67	11.68

#### Analysis of glacier spatial variation characteristics under different slopes

Table 7 reveals the significant regulatory effect of slope on glacier area change rates. In regions with slopes less than 10°, the glacier area decreased from 47,890.13 m^2^ in 2000 to 46,478.88 m^2^ in 2024, representing a reduction of only 3.0%. This suggests that the relatively gentle terrain, characterized by thicker snow cover and higher basal stability, leads to slower melting rates. Furthermore, a brief recovery in glacier area was observed between 2015 and 2020, although the overall trend remains downward. When the slope ranges from 15°to 30°, the glacier area change rate is moderate compared to other slope intervals. Specifically, at slopes of 25°–30°, the glacier area decreased from 5,583.73 m^2^ to 3,990.32 m^2^, which is attributed to the combined effect of surface ablation and basal sliding. For slopes greater than 45°, the glacier area experienced the most drastic change, shrinking sharply from 790.95 m^2^ in 2000 to 307.59 m^2^ in 2024, a decline of 61.11%. This can be attributed to the gravitational collapse of ice masses on steep slopes and the thin snow cover that limits replenishment through snowfall. In the 30–35° range, the glacier area showed some fluctuations over time, but the overall trend still indicated a continuous decline.

**Table 7:** Statistical table of glacier area (m^2^) at different slopes.

Year	Slope/^∘^								
	<10	10–15	15–20	20–25	25–30	30–35	35–40	40–45	>45
2000	47,890.13	15,018.63	9,952.79	7,476.36	5,583.73	4,011.25	2,354.02	1,082.85	790.95
2005	47,428.01	14,644.83	9,632.80	7,303.78	5,589.63	4,127.01	2,459.44	1,117.93	857.08
2010	46,487.84	14,398.81	9,411.82	7,081.53	5,286.21	3,790.11	2,284.95	1,088.07	843.26
2015	45,641.40	13,891.62	8,942.13	6,593.52	4,819.01	3,427.24	2,061.56	956.84	652.39
2020	46,650.74	13,840.12	8,658.60	6,161.58	4,329.30	2,914.61	1,610.70	681.78	374.98
2024	46,478.88	13,600.33	8,387.98	5,860.78	3,990.32	2,585.39	1,371.67	548.67	307.59
2000–2024 area change rate/%	2.95	9.44	15.72	21.61	28.54	35.55	41.73	49.33	61.11

#### Analysis of spatial variations in glacier extent under different aspects

According to Table 8, the rate of glacier area change varies significantly among different slope aspects. Since slope aspect determines the amount of solar radiation received by a surface, this spatial differentiation pattern is closely related to variations in solar energy input across aspects. Glaciers on north-facing and northwest-facing slopes experienced the greatest area reductions, decreasing by 19.71% and 20.81%, respectively. In contrast, the south-facing glaciers showed a smaller reduction-from 10,197.60 m^2^ in 2000 to 9,543.51 m^2^ in 2024, a decline of only 6.41%. This spatial differentiation suggests that glaciers on south-facing slopes exhibit stronger stability under current climatic conditions. Similarly, glaciers on southwest-facing slopes decreased from 9,086.51 m^2^ to 8,404.60 m^2^, a 7.50% reduction. The retreat of east-facing and southeast-facing slopes fell between these two categories, with area reductions of 8.9% and 6.7%, respectively. This is associated with their moderate exposure to sunlight and relatively efficient snow redistribution. Glaciers on flat slopes exhibit relatively small changes in area. This is primarily attributed to the fact that flat terrain favors the long-term coverage and preservation of snow. A thicker snow layer can effectively block solar heat, thereby reducing glacial ablation.

**Table 8:** Statistical table of glacier area (m^2^) at different aspects.

Year	Aspects								
	Flat	Northern	North- eastern	Eastern	South- eastern	Southern	South- western	Western	North- western
2000	677.96	13,304.91	13,257.83	13,050.67	11,723.01	10,197.60	9,086.51	10,884.98	11,977.24
2005	670.76	13,089.05	13,079.73	12,781.62	11,533.27	10,126.55	9,241.52	10,937.04	11,700.96
2010	661.91	12,938.98	12,585.36	12,385.88	11,243.40	9,819.84	8,813.38	10,590.56	11,633.29
2015	643.69	12,343.27	12,082.31	11,777.86	10,760.13	9,455.35	8,524.60	10,290.41	11,108.07
2020	647.69	11,368.67	11,965.22	11,965.22	10,925.51	9,527.86	8,496.67	10,073.29	10,252.25
2024	631.80	10,682.41	11,813.00	11,896.13	10,940.12	9,543.51	8,404.60	9,734.71	9,485.32
2000–2024 area change rate/%	6.81	19.71	10.90	8.85	6.68	6.41	7.50	10.57	20.81

### Analysis of glacier response to climate change

Glacier area changes are influenced by multiple factors, among which climate is a key driver. Specifically, temperature and precipitation are considered the most critical climatic variables influencing glacier dynamics (Baig, Khan & Din, 2018; Chen et al., 2018; Liu et al., 2020; Zhang et al., 2022). Due to the inherent complexity of glacier systems and their unique interactions with the climate system, the impact of climate change on glaciers is not immediate, but rather exhibits a lagged response (Chen et al., 2025; Zhang et al., 2025a; Zhang et al., 2025b; Wang et al., 2023). To quantitatively determine the lag time in glacier response to climate change, this study analyzes the relationship between mean annual temperature, total precipitation and glacier area change. Lagged correlation analysis and the cross-correlation function (CCF) method are employed to calculate the correlation coefficients under different lag periods (0–8 years), as shown in Table 9.

**Table 9:** Correlation strength of annual mean temperature and total precipitation with glacier variations under different lag years.

	1-year lag	2-year lag	3-year lag	4-year lag	5-year lag	6-year lag	7-year lag	8-year lag
—r— of mean annual temperature	0.34	0.41	0.35	0.36	0.97	0.94	0.72	0.53
—r— of total precipitation	0.32	0.21	0.10	0.22	0.81	0.77	0.75	0.76

The results indicate that when the lag period is five years, the Pearson correlation coefficients between glacier area change and mean annual temperature and total precipitation reach their highest absolute values (—r— = 0.97 and 0.81, respectively). This suggests that the impact of climate change on glacier area is most pronounced approximately five years after the climatic variations occured. To clearly illustrate the corresponding relationship, this study adopts a pairing method of “glacial time corresponding to climatic time (5 years in advance)”, and uses glacial time as a representative in the subsequent analysis section. For instance, the glacier period 2000-2005 corresponds to the climate period 1995–2000, 2005–2010 corresponds to 2000–2005, and so on, with 2020–2024 corresponding to 2015–2019. All subsequent correlation analyses based on temporal intervals were conducted and interpreted according to this correspondence scheme.

#### Analysis of the driving effect of mean annual temperature on glacier changes

**Figure 5:**
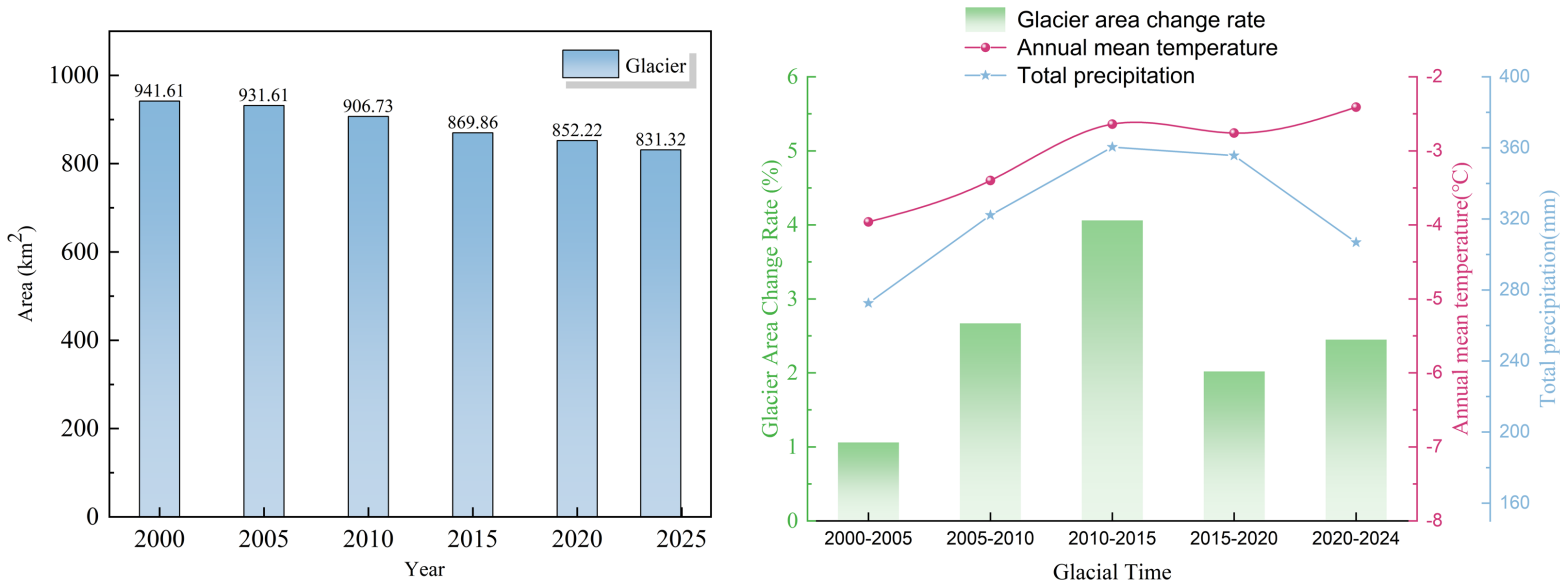
Map of glacier area changes in relation to annual mean temperature and total precipitation for each period.

Based on Fig. 5 and Table 10, it is evident that the glacier area in the study region exhibits a general declining trend, while the mean annual temperature shows a fluctuating upward trend. The glacier area is negatively correlated with the annual average temperature. During different periods, the glacier area has continuously reduced, while the annual average temperature has consistently increased—rising from −3.96 °C during the glacial period of 2000–2005 to −2.41 °C during 2020–2024. This period not only saw the smallest change in glacier area (a reduction of only 10 km^2^), but also corresponded to the lowest average temperature (−3.96 °C). In contrast, the period from 2010 to 2015 experienced the largest decrease in glacier area, with a reduction of 36.87 km^2^, accompanied by a relatively higher average temperature of −2.64 °C. Overall, glaciers in the study area appear to be highly sensitive to temperature variations, exhibiting a inverse relationship between glacier area and mean annual temperature-glacier area tends to decrease with rising temperatures and increase with falling temperatures.

**Table 10:** Glacier area and climate change trends for the periods 2000–2024.

Glacial time	Glacier area change/km^2^	Glacier area change rate/%	Mean annual temperature /°C	Total precipitation/mm
2000–2005	−10.00	1.06	−3.96	272.62
2005–2010	−24.88	2.67	−3.40	322.12
2010–2015	−36.87	4.06	−2.64	360.48
2015–2020	−17.64	2.02	−2.76	355.66
2020–2024	−20.90	2.45	−2.41	306.80

#### Analysis of the driving effect of total precipitation on glacier changes

According to Fig. 5 and Table 10, the glacier area shows a general declining trend, while total precipitation displays a fluctuating upward trend. The increase rate of precipitation is lower than that of mean annual temperature. The average glacier area continuously decreased acrossing the study period, whereas the total precipitation increased from 272.62 mm (the glacier period 2000–2005) to 306.80 mm (the glacier period 2020–2024). Overall, despite the rise in precipitation, glacier area continued to decline, indicating that the supply from precipitation was insufficient to offset the strong ablation caused by rising temperatures.

## Discussion

### The implementation and assessment of NDI-RF

In this study, the NDI-RF method was used to achieve accurate extraction of glacier boundaries in the Geladandong region, yielding a Kappa coefficient, OA, F1-score, Recall, and Precision of 0.92, 0.94, 0.92, 0.88, and 0.93, respectively, all outperforming the NDSI-based method and the standard Random Forest model. Compared with remote sensing index methods and conventional Random Forest model, the NDI-RF method strikes a balance between automation and classification accuracy, significantly improving efficiency in large-scale glacier monitoring.

### The topographical regulation of glacier area changes in the Geladandong

The results indicate that over the past 25 years, glaciers in the Geladandong region have exhibited an average annual area change rate of 0.44%, consistent with the retreat trends observed in other glacierized regions of the Tibetan Plateau (Dou et al., 2023; Wang et al., 2020a; Wang et al., 2020b; Shrivas et al., 2025). However, the annual area change rate in the Geladandong glaciers is lower than that of some glaciers in other segments of the Himalayas. For instance, Sun et al. (2025) reported that glaciers in the Parong Zangbo River basin (southeastern Himalayas) experienced an annual area change rate of 1.01% between 1987 and 2023. Li et al. (2019) found that glaciers in the Hala Lake basin decreased by 0.94 km^2^ per year from 1986 to 2015, whereas in this study, glaciers in Geladandong decreased by 4.48 km^2^ per year from 2000 to 2015, indicating a higher sensitivity to climate change in the Geladandong region.

The most significant glacier area change in our study area occurred below 5,500 m, aligning with findings in the Bolivian eastern Cordillera (Veettil et al., 2018). This consistency underscores the particular vulnerability of low-elevation glaciers to global warming. Notably, glaciers in the study area not only show an overall shrinking trend but also exhibit spatial variability in area change rate, suggesting that glacier retreat is strongly influenced by topographic conditions (Robson et al., 2018). Previous studies (Mir, 2018) have shown that steeper slopes accelerate the glacier retreat. Similarly, glaciers on steep slopes experienced a 61.1% reduction in area, confirming the amplifying effect of steep topography on ablation rate. In addition, south-facing glaciers show slower area reduction compared to north-facing ones due to the ablation–accumulation balance mechanism, consistent with the findings of Zhang et al. (2024a) and Zhang et al. (2024b) for glaciers in Geladandong, further supporting the regulatory role of slope aspect on glacier stability.

### Climate-driven mechanisms of glacier change

This study reveals the complexity of climate-driven mechanisms affecting glaciers. Glacier area shows a general declining trend over time, exhibiting a negative correlation with rising mean annual temperature, while the relationship with precipitation changes is relatively weak. These findings are consistent with the conclusion of Piroton et al. (2017) that global warming drives environmental changes in high-mountain glaciers. Rising temperatures increase the energy input to glacier surfaces, accelerating ice and snow ablation (Ahmad et al., 2021; Purushottam, Aparna & Avtar, 2019; Zhang et al., 2018).

The meteorological data used in this study primarily come from the Tuotuohe station, where the long-term mean temperature is approximately −4.1 °C, whereas the long-term mean temperature in the Geladandong region ranges from −9 to −10 °C (Chao et al., 2017). This indicates that while data from a single station can reflect general regional climate trends, it may underestimate local climate variability and its impact on glacier evolution, revealing certain spatial limitations. Future research will integrate higher-resolution, multi-station meteorological observations and reanalysis data to further quantify the independent effects of temperature and precipitation on glacier changes, thereby deepening the understanding of climate-driven mechanisms. Additionally, the influence of precipitation on glaciers is more complex. The precipitation data do not differentiate between precipitation types. On one hand, snowfall can supply mass to glaciers, enhance surface albedo, and mitigate ablation to some extent (Fyffe et al., 2025). On the other hand, rainfall can reduce surface reflectivity and release latent heat, accelerating glacier retreat (Zhao et al., 2024). Future work will analyze changes in snowfall proportion using precipitation phase data to more comprehensively reveal how precipitation structure affects glaciers in the Geladandong region.

### Limitations and future scope

At present, there remains considerable scope for development in employing the NDI-RF method to extract glacier boundaries. Firstly, the accuracy assessment relied on The Second Glacial Catalogue Dataset of China (Version 1.0), which has certain temporal limitations. Secondly, this study used Landsat imagery for glacier boundary extraction, and its spatial resolution may limit the identification of small or narrow glaciers. Finally, although the NDI-RF method performed well in this experiment, the accuracy evaluation was based on the entire glacier area and did not specifically verify debris-covered zones, making it difficult to quantify the specific contribution of NDWI to accuracy improvement. To address the aforementioned limitations, further work will conduct experiments using the Randolph Glacier Inventory (RGI) v7.0 as the validation benchmark, aiming to obtain assessment results with enhanced timeliness and broader regional applicability. Concurrently, high-resolution imagery such as Sentinel-2 may be incorporated within the same algorithmic framework for comparative analysis, thereby testing the robustness of the NDI-RF method across varying data resolutions. Furthermore, more targeted validation schemes should be designed for typical complex regions to systematically assess the applicability of the NDI-RF method across diverse glacial environments.

## Conclusion

This study, based on Landsat series satellite remote sensing imagery, developed a glacier identification method (NDI-RF) that integrates remote sensing indices with machine learning. By removing the influence of glacial lakes, the method significantly improved the accuracy and stability of glacier boundary extraction. The proposed method was effectively applied to extract glacier boundaries in the Geladandong region over the past 25 years. The spatiotemporal characteristics of glaciers in the Geladandong region and their response to climatic drivers were examined with respect to topographic and climatic elements. The main conclusions are as follows:

(1) In this study, the NDI-RF method outperformed both the NDSI method and the Random Forest model, achieving a Kappa coefficient of 0.92, an overall accuracy of 0.94, an F1-score of 0.92, a recall of 0.88, and a precision of 0.93. These results confirm the method’s enhanced capability in mitigating spectral confusion caused by ice lakes and thin ice.

(2) Between 2000 and 2024, glaciers in the Geladandong region experienced widespread area reduction, with a total loss of 110.29 km^2^ and an average annual rate of 0.47%. The period from 2010 to 2015 was marked by the most significant shrinkage, recording a decrease of 36.87 km^2^ and the highest absolute annual rate of area change. Topographic analysis further revealed that glacier loss was most pronounced at elevations below 5,250 m, on slopes steeper than 45°, and in northwest-facing areas.

(3) Over the past 25 years, the average annual temperature and total precipitation in the study area have generally shown a fluctuating upward trend. Glacier area changes exhibit a negative correlation with mean annual temperature.

Looking forward, future studies could apply the NDI-RF method to broader glacial regions and integrate higher-resolution remote sensing data with multi-source climate datasets to further elucidate the heterogeneous response mechanisms of glaciers to climate change.

##  Supplemental Information

10.7717/peerj.20804/supp-1 Supplemental Information 1 Random Forest Algorithm

10.7717/peerj.20804/supp-2 Supplemental Information 2 DEM data for the study area

10.7717/peerj.20804/supp-3 Supplemental Information 3 Meteorological data

10.7717/peerj.20804/supp-4 Supplemental Information 4 The second glacial catalogue dataset of China (version 1.0)

10.7717/peerj.20804/supp-5 Supplemental Information 5 2000 Remote Sensing Imagery Please use professional software such as ENVI or ArcMap to open. Standard image viewers may display an error indicating the file cannot be opened.

10.7717/peerj.20804/supp-6 Supplemental Information 6 2005 Remote Sensing Imagery Please use professional software such as ENVI or ArcMap to open. Standard image viewers may display an error indicating the file cannot be opened.

10.7717/peerj.20804/supp-7 Supplemental Information 7 2007 Remote Sensing Imagery Please use professional software such as ENVI or ArcMap to open. Standard image viewers may display an error indicating the file cannot be opened.

10.7717/peerj.20804/supp-8 Supplemental Information 8 2010 Remote Sensing Imagery Please use professional software such as ENVI or ArcMap to open. Standard image viewers may display an error indicating the file cannot be opened.

10.7717/peerj.20804/supp-9 Supplemental Information 9 2015 Remote Sensing Imagery Please use professional software such as ENVI or ArcMap to open. Standard image viewers may display an error indicating the file cannot be opened.

10.7717/peerj.20804/supp-10 Supplemental Information 10 2020 Remote Sensing Imagery Please use professional software such as ENVI or ArcMap to open. Standard image viewers may display an error indicating the file cannot be opened.

10.7717/peerj.20804/supp-11 Supplemental Information 11 2024 Remote Sensing Imagery Please use professional software such as ENVI or ArcMap to open. Standard image viewers may display an error indicating the file cannot be opened.

## Additional Information and Declarations

### Data Availability

The following information was supplied regarding data availability: The raw data and code are available in the Supplemental Files. The remote sensing imagery is available at Geospatial Data Cloud platform: https://www.gscloud.cn/sources/accessdata/411?pid=263
The remote sensing imagery is available at Earth Explorer platform of the United States Geological Survey: https://earthexplorer.usgs.gov/
- Latitude and longitude coordinates of the study area required to download data: 90°00′E–92°00′E, 32°50′N–33°50′N. The second glacial catalogue dataset of China (version 1.0) is available at National Cryosphere Desert Data Center: https://www.ncdc.ac.cn/portal/metadata/6d44fd19-64d7-4af1-8e81-5fa717585b5b
The DEM data is available at National Aeronautics and Space Administration: https://science.nasa.gov/resource/asters-global-digital-elevation-model
The meteorological data is available at China Meteorological Data Service Centre:
https://data.cma.cn/dataService/cdcindex/datacode/A.0012.0001/show_value/normal.html.
