# Supplementary figures and images for: Glacier boundary extraction and spatiotemporal variation analysis in Geladandong region
Source: PeerJ. 2026 Feb 18;14:e20804. doi: 10.7717/peerj.20804 (PMC12924652; doi:10.7717/peerj.20804)

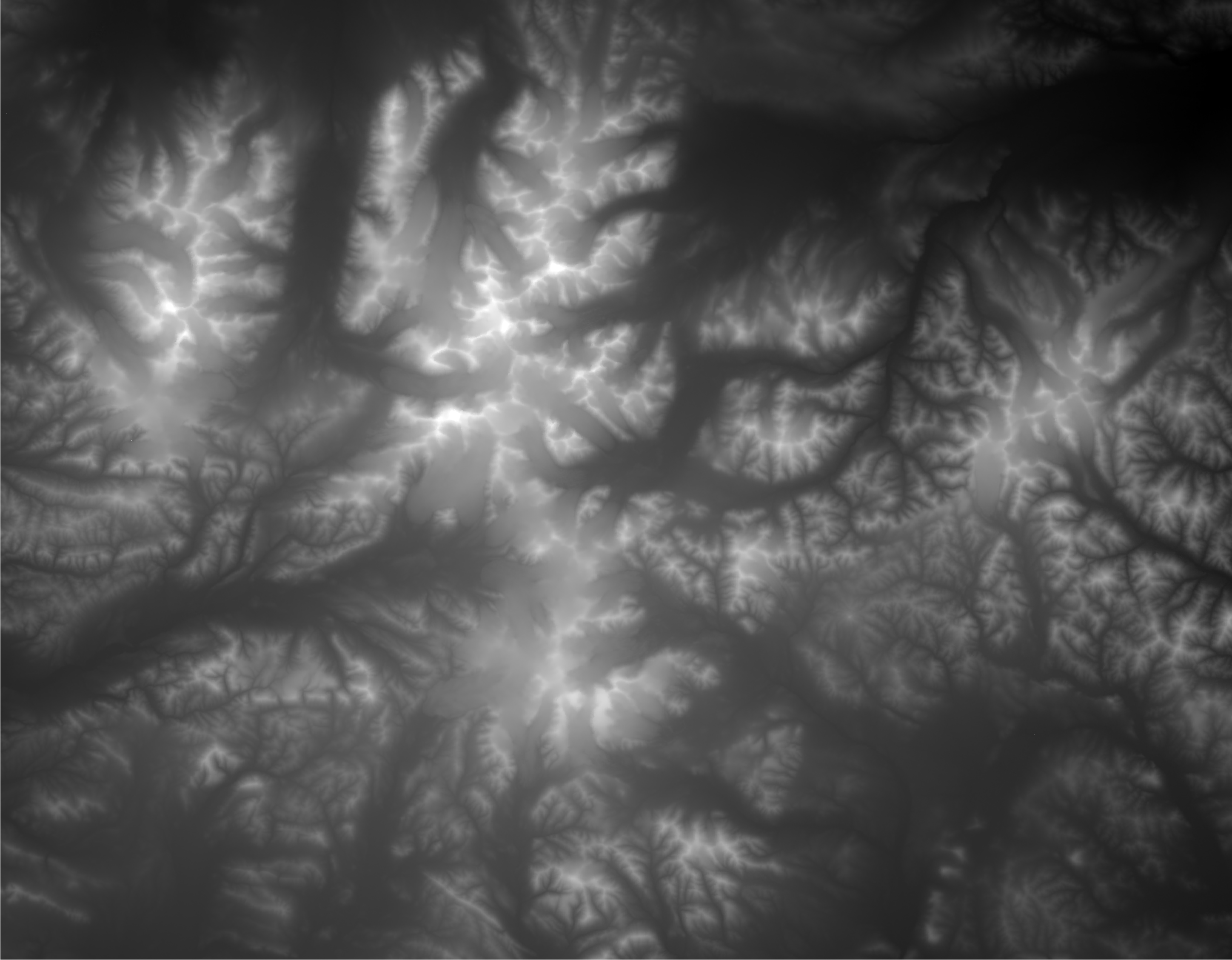

Supplement: Supplemental Information 2 [file peerj-14-20804-s002.zip › DEM/dem.tif]
